# *Gpx4* Deletion‐Mediated Macrophage Ferroptosis Alleviates Obesity‐Associated Insulin Resistance

**DOI:** 10.1096/fj.202503596R

**Published:** 2026-01-12

**Authors:** Suhua Wu, Juan Peng, Xiaodong Wang, Hong Gao, Huangrui Fang, Shiyi Hu, Qiufen Wei, Yuanye Dang, Haixia Tu, Mingyan Zhu, Jianye Peng, Yumiao Liu, Gaofeng Zeng, Xiaoyan Dai

**Affiliations:** ^1^ Department of Cardiovascular Medicine, the Second Affiliated Hospital University of South China Hengyang China; ^2^ The Fifth Affiliated Hospital, Guangdong Province & NMPA & State Key Laboratory, School of Pharmaceutical Sciences Guangzhou Medical University Guangzhou China; ^3^ Key Laboratory for Arteriosclerology of Hunan Province, Hunan International Scientific and Technological Cooperation Base of Arteriosclerotic Disease, School of Basic Medical Sciences, Hengyang Medical School, Institute of Cardiovascular Disease University of South China Hengyang China; ^4^ Department of Research, the Second Affiliated Hospital University of South China Hengyang China; ^5^ Clinical Research Institute, the Second Affiliated Hospital University of South China Hengyang China

**Keywords:** ferroptosis, GPX4, insulin resistance, macrophage, obesity

## Abstract

Obesity has become a global epidemic and a major contributor to the development of Type 2 diabetes (T2D) through the promotion of insulin resistance. Emerging evidence has shown that GPX4 expression is reduced in macrophages under hyperglycemic conditions; however, the involvement of macrophage‐specific GPX4 in obesity‐associated insulin resistance remains unclear. We generated macrophage‐specific *Gpx4* knockout (*Gpx4* ^Mac‐KO^) mice and subjected both *Gpx4* ^Mac‐KO^ and littermate *Gpx4* ^fl/fl^ mice to a high‐fat diet (HFD) for 16 weeks. Metabolic parameters, adipose tissue morphology, hepatic lipid accumulation, and free fatty acid (FFA) metabolism were assessed. The results showed that macrophage‐specific deletion of *Gpx4* attenuated HFD‐induced obesity and improved insulin sensitivity in mice in vivo. *Gpx4*‐deficient mice exhibited lower levels of systemic inflammation, reduced adipocyte hypertrophy, and diminished hepatic steatosis. Deficiency of *Gpx4* in macrophages affects FFA metabolism by regulating the expression of FFA breakdown‐related genes, such as *C/EBP‐α*, *PPARγ*, *ATGL*, *Fabp4*, and/or *LPL,* in white adipose tissue and the liver. These beneficial metabolic effects seemed to be associated with enhanced macrophage ferroptosis, suggesting a mechanistic link between *Gpx4* deficiency, ferroptosis, and the alleviation of obesity‐associated insulin resistance. Our findings identify macrophage GPX4 as a key mediator of obesity‐induced insulin resistance and metabolic malfunction. Targeting macrophage GPX4 may represent a promising therapeutic strategy for the treatment of T2D.

## Graphical abstract

Macrophage‐specific loss of *Gpx4* induces ferroptosis, thereby alleviating high‐fat diet (HFD)‐induced obesity, insulin resistance, and metabolic dysfunction, and conferring systemic metabolic protection in type 2 diabetes (T2D).

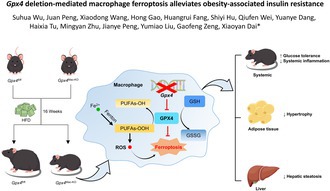

**Abbreviations**
ATGL: adipose triglyceride lipase
BAT: brown adipose tissue
BMDMs: bone marrow‐derived macrophages
DEGs: differentially expressed genes
ELISA: Enzyme‐linked immunosorbent assay
FBS: fetal bovine serum
Fer‐1: Ferrostatin‐1
FFA: free fatty acid
GPX4: Glutathione peroxidase 4
GTT: glucose tolerance tests
H&E: Hematoxylin and eosin
HDL‐C: high‐density lipoprotein cholesterol
HFD: high‐fat diet
HOMA‐IR: homeostatic model assessment of insulin resistance
HSF1: heat shock factor 1
HSL: hormone‐sensitive lipase
ITT: insulin tolerance tests
LDL‐C: low‐density lipoprotein cholesterol
MAFLD: metabolic dysfunction‐associated fatty liver disease
M‐CSF: macrophage colony‐stimulating factor
MGL: monoglyceride lipase
NAFLD: Nonalcoholic fatty liver disease
PA: Palmitic acid
PI3K: phosphoinositide 3‐kinase
PMs: Peritoneal macrophages
qPCR: quantitative polymerase chain reaction
T2D: type 2 diabetes
TG: triglycerides
WAT: white adipose tissue

## 1 Introduction

Obesity is a complex metabolic disorder characterized by excessive energy intake, fat accumulation, and disruption of energy homeostasis [1]. It affects multiple organ systems, including the cardiovascular, hepatic, renal, musculoskeletal, and reproductive systems, contributing to a range of diseases such as T2D, cardiovascular disease, hypertension, stroke, certain cancers, and mental health conditions [2]. A key pathophysiological feature of obesity is insulin resistance, a condition in which insulin‐sensitive tissues (e.g., skeletal muscle, adipose tissue, and liver) exhibit a diminished response to insulin, leading to impaired glucose uptake and hyperglycemia [3]. Chronic, low‐grade inflammation in obese individuals has been identified as a central driver of insulin resistance [4]. Despite extensive research into therapeutic strategies for obesity and related metabolic disorders, significant breakthroughs remain elusive, highlighting the need for novel targets and approaches.

Glutathione peroxidase 4 (GPX4) is a critical selenoenzyme that catalyzes the reduction of lipid hydroperoxides (L‐OOH) to their corresponding alcohols (L‐OH), using glutathione as a substrate, thereby protecting cells from oxidative damage [5]. As a key regulator of redox homeostasis, GPX4 has been extensively studied in the context of oxidative stress, lipid metabolism, apoptosis, and ferroptosis, a regulated, iron‐dependent form of cell death driven by lipid peroxidation [6, 7]. Recent studies have begun to elucidate its role in metabolic diseases. For instance, in obese mice, reduced GPX4 activity in the liver promotes ferroptosis and inflammation, while inhibition of ferroptosis with ferrostatin‐1 restores GPX4 levels, shifts macrophage polarization from M1 to M2, reduces oxidative stress, and protects against metabolic liver damage [8]. Furthermore, GPX4 downregulation in adipose tissue has been implicated in vagal nerve ferroptosis and autonomic dysfunction in diabetes, via suppression of the NRF2‐GPX4 axis [9]. In the context of diabetes, GPX4 deficiency can exacerbate ferroptosis, potentially contributing to β‐cell dysfunction and other complications [10]. These findings suggest a strong link between GPX4 and obesity‐associated metabolic disorders. Macrophages play a central role in the development of insulin resistance, acting as key mediators of inflammation within metabolic tissues such as adipose tissue, liver, and skeletal muscle [11]. However, the specific role of macrophage‐derived GPX4 in obesity‐induced insulin resistance remains largely undefined.

In this study, using macrophage‐specific *Gpx4* knockout mice (*Gpx4* ^Mac‐KO^), we found that deletion of macrophage *Gpx4* reduced weight gain, improved insulin sensitivity, alleviated adipose tissue and liver abnormalities, and enhanced free fatty acid metabolism. The benefits appear to be mediated by ferroptosis in *Gpx4*‐deficient macrophages. These findings suggest that targeting macrophage GPX4 could offer a novel therapeutic approach for T2D.

## 2 Materials and Methods

### 2.1 Mouse Model

*Gpx4* ^fl/fl^ mice (STOCK *Gpx4* ^tm1.1Qra^/J, 027964) were purchased from Jackson Laboratory and crossed with *Lyz2*‐Cre transgenic mice (B6.129P2‐Lyz2^tm1(cre)Ifo/J^, 004781, Jackson Laboratory) to generate macrophage‐specific *Gpx4* knockout (*Gpx4* ^Mac‐KO^) mice. All mice were on a C57BL/6J genetic background. Both *Gpx4* ^fl/fl^ (wild‐type controls) and *Gpx4* ^Mac‐KO^ mice were included in this study.

Mice were housed under controlled conditions (temperature: 22°C–26°C; humidity: 40%–70%; 12 h light/dark cycle) with ad libitum access to food and water. A high‐fat diet (HFD; 60% kcal from fat; D12492) was purchased from Research Diets (USA). Eight‐week‐old male *Gpx4* ^fl/fl^ mice (*n* = 8) and *Gpx4* ^Mac‐KO^ mice (*n* = 8) were fed an HFD for 16 weeks. In parallel, 8‐week‐old male *Gpx4* ^fl/fl^ mice (*n* = 7) and *Gpx4* ^Mac‐KO^ mice (*n* = 8) were fed a standard chow diet for 16 weeks. All enrolled mice completed the study, and no attrition occurred. All animal experiments were approved by the Animal Protection Committee of Guangzhou Medical University (No. 2019–016).

### 2.2 Cell Culture

Primary bone marrow‐derived macrophages (BMDMs) were prepared by flushing bone marrow from the femur and tibia. Cells were cultured in complete RPMI‐1640 medium containing 10% fetal bovine serum (FBS) and recombinant mouse macrophage colony‐stimulating factor (M‐CSF, 25 ng/mL; #14‐8983‐80, eBioscience, Invitrogen) for 5 days at 37°C in 5% CO_2_.

Peritoneal macrophages (PMs) were collected 3 days after intraperitoneal injection of 4% thioglycolate (1.5 mL/mouse; BD Biosciences). The peritoneal cavity was lavaged with 8 mL cold PBS containing 10 mM EDTA and 10% FBS. Cells were plated in RPMI‐1640 with 10% FBS at 1.0 × 10^6^ cells/mL, incubated for 3 h at 37°C, and non‐adherent cells were removed. Adherent macrophages were used for subsequent experiments.

### 2.3 Glucose and Insulin Tolerance Tests

For glucose tolerance tests (GTT), mice were fasted for 16 h and injected intraperitoneally with glucose (1 g/kg body weight; 25% D‐glucose, #G7528; Sigma‐Aldrich). Blood glucose levels were measured from tail vein blood at 0, 15, 30, 60, 90, and 120 min.

For insulin tolerance tests (ITT), mice were fasted for 4 h and administered insulin intraperitoneally (1.5 U/kg body weight; #I9278; Sigma‐Aldrich). Blood glucose was similarly measured. The homeostatic model assessment of insulin resistance (HOMA‐IR) was calculated as:

$$\begin{array}{c} \text{HOMA}-\mathrm{IR}=\\ \left[\text{Fasting insulin}\;\left(\mathrm{mIU}/L\right)\times \text{Fasting glucose}\;\left(\text{mmol}/L\right)\right]/22.5 \end{array}$$

### 2.4 Western Blot Analysis

Protein lysates from mouse BMDMs, PMs, WAT, liver, skeletal muscle, and kidney were prepared using RIPA buffer and quantified using a BCA protein assay. Equal amounts (50 μg) were separated by 10%–12% SDS‐PAGE, transferred to nitrocellulose membranes (#66485; Pall), and blocked with 5% BSA. Membranes were incubated overnight at 4°C with primary antibodies (1:1000), followed by HRP‐conjugated secondary antibodies (1:5000) for 1 h at room temperature. Blots were visualized with Immobilon ECL substrate (#P90719; Millipore) and quantified using ImageJ. Antibodies against phospho‐Akt (Ser473) (#9271, Cell Signaling Technology), Akt (#9272; Cell Signaling Technology), *Gpx4* (#PA579321; Thermo Fisher Scientific), GAPDH (#sc‐137 179; Santa Cruz Biotechnology, INC), and α‐Tubulin (#sc‐32 293, Santa Cruz Biotechnology Inc) were used. GAPDH and α‐Tubulin were used as loading controls.

### 2.5 Enzyme‐Linked Immunosorbent Assay (ELISA)

Plasma concentrations of insulin and inflammatory cytokines were quantified using the following kits: Mouse Insulin ELISA (#10‐1247‐01; Mercodia), TNF‐α (#MTA00B; R&D Systems), and IL‐1β (#MLB00C; R&D Systems). Absorbance was measured at 450 and 540 nm. Concentrations were calculated according to standard curves.

### 2.6 Lipid Analysis

Plasma levels of triglycerides (TG), total cholesterol, high‐density lipoprotein cholesterol (HDL‐C), and low‐density lipoprotein cholesterol (LDL‐C) were measured using a fully automated biochemical analyzer (Hitachi 7600–020). For tissue lipid quantification, WAT and liver samples were homogenized in 100% ethanol, centrifuged, and supernatants analyzed using assay kits (Jiancheng Bioengineering Institute, Nanjing, China).

### 2.7 RNA Extraction and Quantitative Polymerase Chain Reaction (qPCR)

Total RNA was extracted from mouse WAT, liver, and skeletal muscle using AG RNAex Pro reagent (#AG21101; Accurate Biology). cDNA was synthesized from 1 μg RNA using the Evo M‐MLV RT Premix (#AG11601), and qPCR was performed with SYBR Green Pro Tap HS qPCR Kit (#AG1701; Accurate Biology). Primer sequences are listed in Table S1.

### 2.8 Histological Analysis

Adipose tissue and liver samples were fixed in 4% paraformaldehyde, embedded in paraffin, sectioned at 4 μm, and stained with hematoxylin and eosin (H&E). Fat cell area was quantified using ImageJ by measuring at least 300 cells per group. Immunohistochemical staining for F4/80 was performed using anti‐F4/80 antibody (#30325 T, Cell Signaling Technology, 1:200). Frozen liver sections (6 μm, OCT embedded) were stained with Oil Red O.

### 2.9 Lipidomics Analysis

White adipose tissue (WAT) and liver samples from *Gpx4* ^fl/fl^ and *Gpx4* ^Mac‐KO^ mice were collected and analyzed in randomized order. Approximately 20 mg of tissue was homogenized in 200 μL of water using four grinding beads for 1 min. Ten microliters of internal standard solution (heptadecanoic acid, FFA 19:0, 12.5 μg/mL) was added, followed by vortexing. Lipids were extracted by adding 800 μL of chloroform: methanol (2:1, v/v), vortexed, incubated for 30 min, and centrifuged at 13 000 × *g* for 20 min. The lower organic phase was collected, and 100 μL was dried under nitrogen and reconstituted in 50 μL of solvent for analysis.

Chromatographic separation was performed on a Waters XBridge Peptide BEH C18 column (3.5 μm, 2.1 × 100 mm) with a Phenomenex C18 guard column using an Eksigent LC100 system. Mobile phases were 10 mM ammonium acetate with 0.1% formic acid in water (A) and 10 mM ammonium acetate with 0.1% formic acid in acetonitrile: isopropanol (49.95:49.95, v/v) (B). The gradient program was: 0.01 min, 65% A/35% B; 2 min, 20% A/80% B; 8 min, 0% A/100% B; 9 min, 65% A/35% B; 11 min, 65% A/35% B. Column temperature was maintained at 50°C, flow rate 0.4 mL/min, injection volume 2 μL.

Mass spectrometry was performed on an AB SCIEX Triple TOF 5600 in negative ion mode. Full‐scan spectra were acquired over *m/z* 100–1200, and information‐dependent acquisition (IDA) was performed over *m/z* 50–1200. Source parameters: GS1/GS2, 50; curtain gas, 30; source temperature, 550°C; ion spray voltage, −4500 V; declustering potential, −80 V; collision energy, −10 V for TOF‐MS and −35 V for IDA.

Fatty acids were identified using PeakView 1.2 and quantified with MultiQuant 2.1 software, referencing a standard fatty acid database. Absolute concentrations were calculated using standard curves normalized to the internal standard.

### 2.10 RNA‐Sequencing (RNA‐Seq) Analysis

Total RNA from BMDMs of 6–8‐week‐old littermate mice (*Gpx4* ^fl/fl^ and *Gpx4* ^Mac‐KO^) was extracted and submitted to Lianchuan Biotechnology Co. Ltd. (Hangzhou, China) for RNA‐seq. Differentially expressed genes were identified based on adjusted *p*‐value (FDR) < 0.05 and |log_2_(FC)| ≥ 1.

### 2.11 Quantification of Glutathione (GSH)

A GSH assay kit was purchased from Njjcbio (Jiangsu, China; A006‐2‐1). BMDMs cultured for 5 days were collected and subjected to ultrasonic disruption to obtain cell lysates. The lysates were mixed with Reagent 1 at a 1:1 ratio and centrifuged at 3500 rpm for 10 min. The resulting supernatants were collected and subsequently incubated with Reagents 2 and 3 according to the manufacturer's instructions for 5 min. Absorbance was measured at 405 nm using a microplate reader.

### 2.12 Quantification of Malondialdehyde (MDA)

A Micro MDA assay kit was purchased from Njjcbio (Jiangsu, China; A003‐2). BMDMs cultured for 5 days were collected and subjected to ultrasonic disruption to obtain cell lysates. The samples were mixed with Reagents 1, 2, and 3 according to the manufacturer's instructions and incubated in a 95°C water bath for 40 min. After incubation, the mixtures were centrifuged at 1800 g for 10 min, and absorbance was measured at 532 nm using a microplate reader.

### 2.13 Immunofluorescence

For immunofluorescence staining of adipose tissues, paraffin‐embedded sections were baked at 55°C for 30 min, followed by deparaffinization and rehydration. Antigen retrieval was performed using an improved citrate antigen retrieval solution (Beyotime, P0083). The sections were then blocked with normal goat serum for 1 h at room temperature and incubated with primary antibodies against F4/80 (1:100; Thermo Fisher Scientific, 14‐4801‐81) and COX‐2 (1:100; Abcam, ab15191) at 4°C overnight. After washing three times with PBS, sections were incubated with appropriate fluorophore‐conjugated secondary antibodies and counterstained with 4′,6‐diamidino‐2‐phenylindole (DAPI) for 1 h at room temperature. Images were acquired using a ZEISS LSM 980 laser scanning confocal microscope.

### 2.14 CCK‐8 Cell Viability Assay

BMDMs were seeded at 8 × 10^3^ cells/well in 96‐well plates and differentiated. Cells were treated with palmitic acid (PA, #P5585; Sigma‐Aldrich) and/or ferrostatin‐1 (Fer‐1, #S7243; Selleck). After treatment, medium was replaced with 100 μL of CCK‐8 solution, incubated for 2–4 h, and absorbance at 450 nm was measured. Cell viability (%) = [(*A*_drug − *A*_blank)/(*A*_control − A_blank)] × 100.

### 2.15 Statistical Analysis

Data are expressed as mean ± SEM from at least three independent experiments. Statistical analyses were conducted using GraphPad Prism 10.1.2. Student's *t* test was used for two‐group comparisons, and one‐way ANOVA with Tukey's post hoc test for multiple groups. Normality was assessed using the Shapiro–Wilk test. *p* < 0.05 was considered statistically significant.

## 3 Results

### 3.1 Macrophage‐Specific *Gpx4* Deficiency Attenuates Diet‐Induced Obesity In Vivo

To investigate the role of macrophage GPX4 in obesity‐induced insulin resistance, we generated *Gpx4* ^Mac‐KO^ mice by crossbreeding *Gpx4* ^fl/fl^ mice with *Lyz2*‐Cre mice (Figure S1A). Genotyping was assessed by PCR (Figure S1B). We validated the efficiency of *Gpx4* deletion in macrophages. qPCR results showed that *Gpx4* mRNA was markedly down regulated in both peritoneal macrophages (PMs) and bone marrow‐derived macrophages (BMDMs) from *Gpx4* ^Mac‐KO^ mice compared to *Gpx4* ^fl/fl^ mice (Figure S1C,D). Consistently, Western blot analysis demonstrated significantly reduced GPX4 protein levels in PMs and BMDMs from *Gpx4* ^Mac‐KO^ mice (Figure S1E,F), confirming efficient deletion of *Gpx4* in macrophages.

**FIGURE 1:**
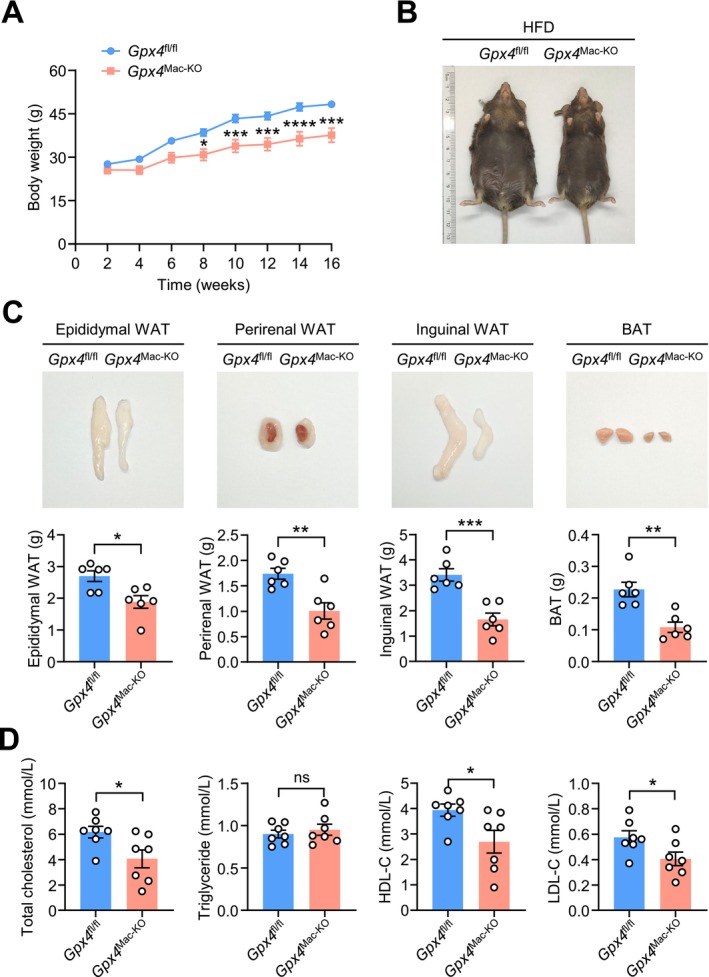
Macrophage‐specific *Gpx4* knockout attenuates diet‐induced obesity. Eight‐week‐old male *Gpx4* ^fl/fl^ and *Gpx4* ^Mac‐KO^ mice were fed on a high‐fat diet (HFD) for 16 weeks. At the end of the experiment, mice were fasted for 16 h prior to sacrifice. The tissues and plasma were collected for further analysis. (A) Body weight gain of chow‐fed *Gpx4* ^fl/fl^ and *Gpx4* ^Mac‐KO^ mice was monitored every 2 weeks from 8 weeks of age to 24 weeks of age (*n* = 8 mice per group). (B) Representative photographs of *Gpx4* ^fl/fl^ and *Gpx4* ^Mac‐KO^ mice (*n* = 8 mice per group). (C) Representative photographs of fat pads (epididymal WAT, perirenal WAT, inguinal WAT, and BAT) (top) and quantification (bottom) in *Gpx4* ^fl/fl^ and *Gpx4* ^Mac‐KO^ mice (*n* = 8 mice per group). (D) Serum lipid profiles (cholesterol, triglycerides, HDL‐C, and LDL‐C) in *Gpx4* ^fl/fl^ and *Gpx4* ^Mac‐KO^ mice (*n* = 7 mice per group). Data are presented as mean ± SEM. ns, not significant. *p < 0.05, **p < 0.01, ***p < 0.001 versus as indicated.

To evaluate the impact of macrophage‐specific *Gpx4* deficiency on obesity, *Gpx4* ^fl/fl^ and *Gpx4* ^Mac‐KO^ mice were fed either a chow diet or a HFD for 16 weeks. No differences in body weight or fat mass were observed between chow‐fed *Gpx4* ^fl/fl^ and *Gpx4* ^Mac‐KO^ mice (Figure S2A,B). However, HFD‐fed *Gpx4* ^Mac‐KO^ mice exhibited significantly lower body weight and reduced fat mass compared to HFD‐fed controls (Figure 1A,B). At 24 weeks of age, HFD‐fed, but not chow‐fed, *Gpx4* ^Mac‐KO^ mice displayed smaller epididymal, perirenal, and inguinal white adipose tissue (WAT) depots, as well as reduced brown adipose tissue (BAT), compared to *Gpx4* ^fl/fl^ mice (Figure 1C; Figure S2C). Additionally, *Gpx4* ^Mac‐KO^ mice had lower serum levels of total cholesterol, low‐density lipoprotein cholesterol (LDL‐C), and high‐density lipoprotein cholesterol (HDL‐C) compared to controls (Figure 1D), while serum triglyceride levels were unchanged between groups (Figure 1D). Collectively, these findings suggest that macrophage‐specific *Gpx4* deficiency mitigates diet‐induced obesity in mice in vivo.

### 3.2 HFD‐Fed *Gpx4* ^Mac^ ^‐KO^ Mice Show Improved Obesity‐Associated Insulin Resistance

**FIGURE 2:**
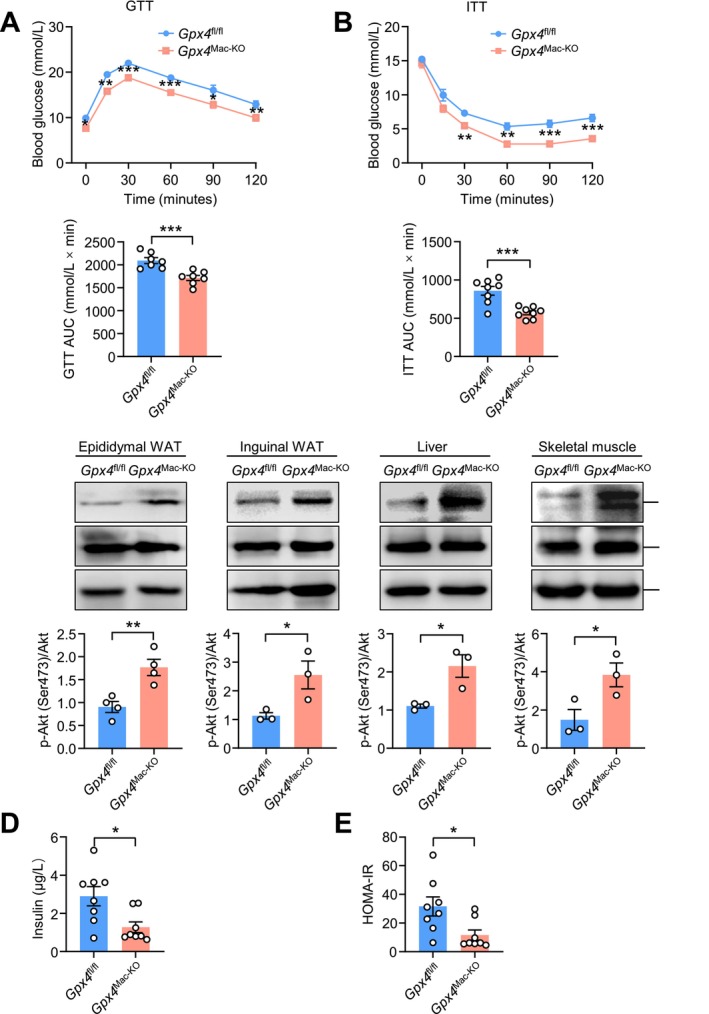
Macrophage‐specific *Gpx4* deficiency alleviates obesity‐induced insulin resistance. (A, B) Glucose tolerance test (GTT) and insulin sensitivity test (ITT) in HFD‐fed *Gpx4* ^fl/fl^ and *Gpx4* ^Mac‐KO^ mice (*n* = 7–8 mice per group). (C) Immunoblot analysis of p‐Akt (Ser473), Akt, and GAPDH in epididymal WAT, inguinal WAT, liver, and skeletal muscle of HFD‐fed *Gpx4* ^fl/fl^ and *Gpx4* ^Mac‐KO^ mice (*n* = 3–4 mice per group). (D) Serum insulin levels in overnight‐fasted HFD‐fed *Gpx4* ^fl/fl^ and *Gpx4* ^Mac‐KO^ mice (*n* = 8 mice per group). (E) Homeostatic model assessment index of insulin resistance (HOMA‐IR) index (*n* = 8 mice per group). Data are presented as mean ± SEM. *p < 0.05, **p < 0.01, ***p < 0.001 versus as indicated.

Obesity is closely associated with the development of insulin resistance. Chronic low‐grade systemic inflammation induced by obesity impairs insulin signaling, disrupts glucose homeostasis, and contributes to insulin resistance and systemic metabolic dysfunction [12, 13]. To investigate the role of macrophage *Gpx4* in obesity related insulin resistance, we performed glucose tolerance tests (GTT) and insulin tolerance tests (ITT) in chow‐ and HFD‐fed *Gpx4* ^fl/fl^ and *Gpx4* ^Mac‐KO^ mice. Under chow‐fed conditions, no significant differences in glucose or insulin tolerance were observed between the two genotypes (Figure S2D,E). However, HFD‐fed *Gpx4* ^Mac‐KO^ mice displayed improved glucose tolerance and insulin sensitivity compared to HFD‐fed *Gpx4* ^fl/fl^ mice (Figure 2A,B).

Since the metabolic effects of insulin are largely mediated through the phosphoinositide 3‐kinase (PI3K)‐AKT signaling pathway, we assessed AKT phosphorylation at Ser473 in epididymal white adipose tissue (eWAT), inguinal WAT (iWAT), liver, and skeletal muscle. Consistent with enhanced insulin sensitivity, HFD‐fed *Gpx4* ^Mac‐KO^ mice exhibited increased levels of phosphorylated AKT (p‐AKT) in these tissues (Figure 2C). Moreover, fasting insulin levels and homeostasis model assessment of insulin resistance (HOMA‐IR) were significantly lower in *Gpx4* ^Mac‐KO^ mice than in *Gpx4* ^fl/fl^ mice (Figure 2D,E), further confirming improved insulin sensitivity. Collectively, these findings demonstrate that deletion of *Gpx4* in macrophages enhances glucose homeostasis and insulin responsiveness under obese conditions.

### 3.3 Macrophage‐Specific *Gpx4* Deficiency Attenuates Inflammation in Mice

Chronic inflammation is a key contributor to obesity and insulin resistance. In obese individuals, the expression of inflammatory mediators in adipose tissue is elevated, which not only disrupts adipose tissue metabolism but also impairs glucose homeostasis, thereby exacerbating obesity [14, 15]. Adipose tissue macrophages are considered major regulators of inflammation in obese adipose tissue [14]. To assess the impact of macrophage‐specific *Gpx4* deficiency on inflammation, we evaluated inflammatory markers in mice.

**FIGURE 3:**
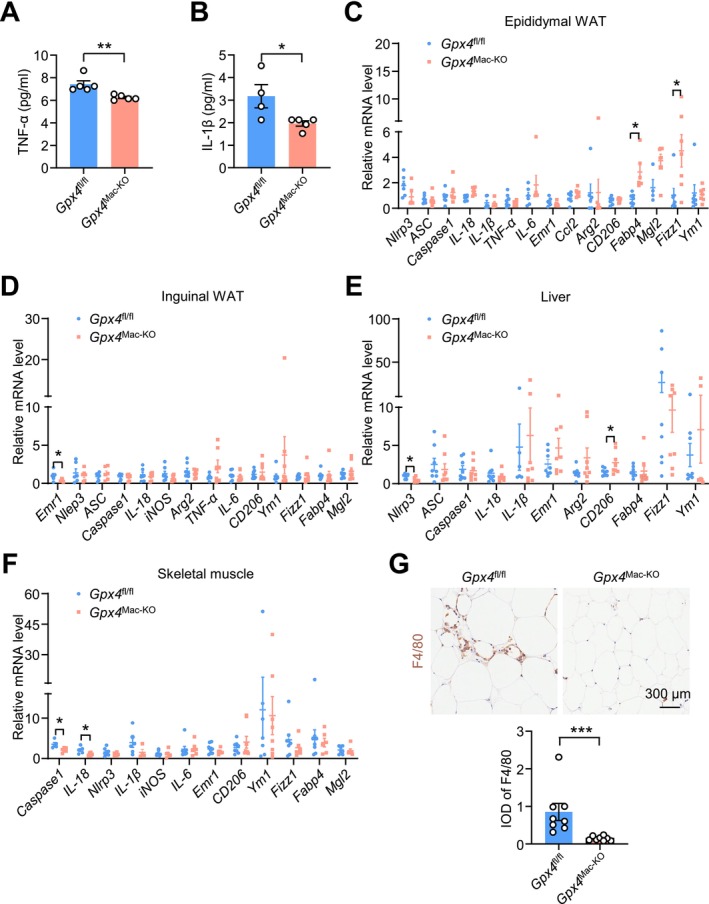
Macrophage‐specific *Gpx4* deficiency reduces systemic inflammation. Eight‐week‐old male *Gpx4* ^fl/fl^ and *Gpx4* ^Mac‐KO^ mice were fed on a HFD for 16 weeks. (A) Serum levels of TNF‐α determined by ELISA (*n* = 5 mice per group). (B) Serum levels of IL‐1β determined by ELISA (*n* = 4 mice for *Gpx4* ^fl/fl^; *n* = 5 for *Gpx4* ^Mac‐KO^). (C) qPCR analysis of mRNA levels of *Nlrp3*, *ASC*, *Caspase1*, *IL‐18*, *IL‐1β*, *TNF‐α*, *IL‐6*, *Emr1*, *Ccl2*, *Arg II*, *CD206*, *Fabp4*, *Mgl2*, *Fizz1*, *Ym1* in epididymal WAT (*n* = 4–7 per group). (D) qPCR analysis of mRNA levels of *Emr1*, *Nlrp3*, *ASC*, *Caspase1*, *IL‐18*, *iNOS*, *Arg II*, *TNF‐α*, *IL‐6*, *CD206*, *Ym1*, *Fizz1*, *Fabp4*, and *Mgl2* in inguinal WAT (*n* = 6–8 per group). (E) qPCR analysis of mRNA levels of *Nlrp3*, *ASC*, *Caspase1*, *IL‐18*, *IL‐1β*, *Emr1*, *ArgII*, *CD206*, *Fabp4*, *Fizz1*, and *Ym1* in liver (*n* = 6–8 per group). (F) qPCR analysis of mRNA levels of *Caspase1*, *IL‐18*, *Nlrp3*, *IL‐1β*, *iNOS*, *IL‐6*, *Emr1*, *CD206*, *Ym1*, *Fizz1*, *Fabp4*, and *Mgl2* in skeletal muscle (*n* = 4–8 per group). (G) Immunohistochemical staining of F4/80 in epididymal WAT (*n* = 8 per group). Data are presented as mean ± SEM. *p < 0.05, **p < 0.01, ***p < 0.001 versus as indicated.

ELISA assays revealed that serum levels of pro‐inflammatory cytokines, including TNF‐α and IL‐1β, were significantly reduced in HFD‐fed *Gpx4* ^Mac‐KO^ mice compared to *Gpx4* ^fl/fl^ mice (Figure 3A,B). In epididymal white adipose tissue (WAT), the expression of the anti‐inflammatory genes *Fabp4* and *Fizz1* was significantly upregulated in *Gpx4* ^Mac‐KO^ mice. The expression of other inflammatory genes showed no significant differences (Figure 3C).

In inguinal WAT, the pro‐inflammatory gene *Emr1* was significantly downregulated in *Gpx4* ^Mac‐KO^ mice compared to controls, while other inflammatory genes remained unchanged (Figure 3D). Similarly, in the liver, *Nlrp3* expression was significantly decreased, and the anti‐inflammatory marker *CD206* was upregulated in *Gpx4* ^Mac‐KO^ mice, with no notable changes in other inflammatory genes (Figure 3E). In skeletal muscle, the expression of pro‐inflammatory genes *Caspase‐1* and *IL‐18* was also reduced in *Gpx4* ^Mac‐KO^ mice (Figure 3F). Furthermore, immunohistochemical staining of epididymal WAT showed decreased macrophage infiltration in *Gpx4* ^Mac‐KO^ mice compared to *Gpx4* ^fl/fl^ mice (Figure 3G).

Together, these findings demonstrate that macrophage‐specific *Gpx4* deficiency alleviates inflammation in mice in vivo.

### 3.4 Macrophage‐Specific *Gpx4* Deficiency Suppresses Adipocyte Hypertrophy in Mice

**FIGURE 4:**
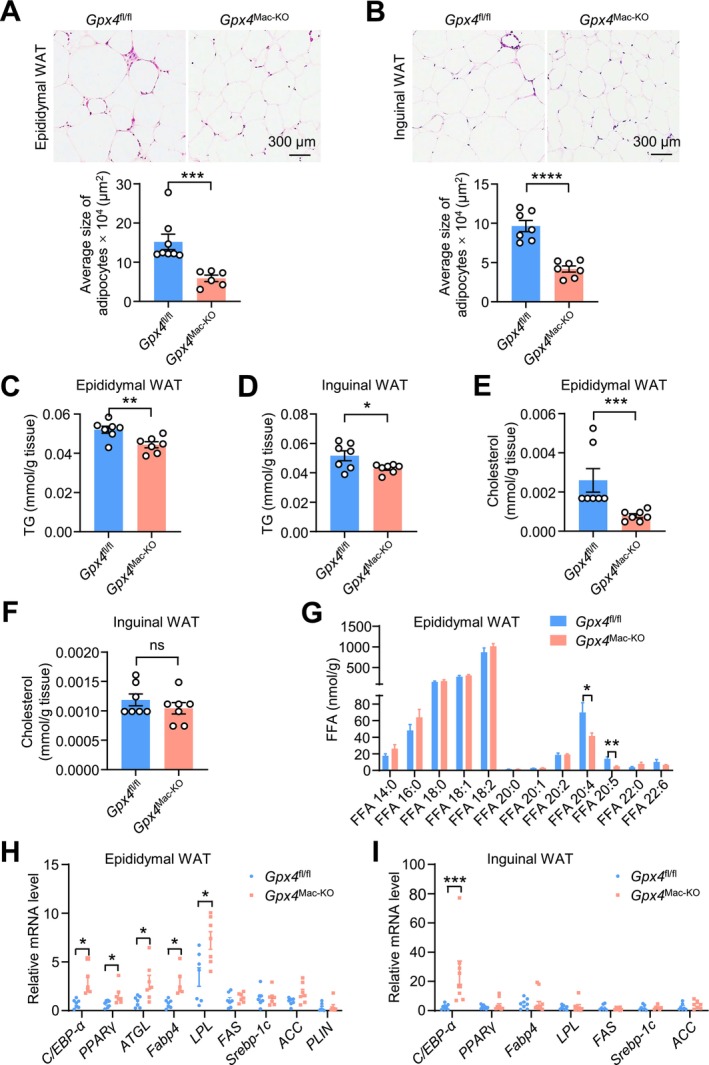
Bone marrow cell‐specific *Gpx4* deficiency limits fat cell hypertrophy. Eight‐week‐old male *Gpx4* ^fl/fl^ and *Gpx4* ^Mac‐KO^ mice were fed on a HFD for 16 weeks. (A, B) H&E staining of epididymal and inguinal WAT tissues (*n* = 6–8 per group). (C, D) The levels of triglycerides (TG) and cholesterol in epididymal (C) and inguinal (D) WAT (*n* = 7 per group). (E, F) The levels of cholesterol in epididymal (E) and inguinal (F) WAT (*n* = 7 per group). (G) Levels of free fatty acids in epididymal WAT isolated from HFD‐fed *Gpx4* ^fl/fl^ and *Gpx4* ^Mac‐KO^ mice (*n* = 7 per group). (H) qPCR analysis of the mRNA levels of *C/EBP‐α*, *PPARγ*, *ATGL*, *Fabp4*, *LPL*, *FAS*, *Srebp‐1c*, *ACC*, and *PLIN* in epididymal WAT (*n* = 6–7 mice per group). (I) qPCR analysis of the mRNA levels of *C/EBP‐α*, *PPARγ*, *Fabp4*, *LPL*, *FAS*, *Srebp‐1c*, and *ACC* in inguinal WAT (*n* = 7–8 mice per group). Data are presented as mean ± SEM. ns, not significant. *p < 0.05, **p < 0.01, ***p < 0.001, ****p < 0.0001 versus as indicated.

To investigate the mechanism underlying reduced adiposity in *Gpx4* ^Mac‐KO^ mice, we assessed adipocyte size in epididymal WAT and inguinal WAT from HFD‐fed *Gpx4* ^fl/fl^ mice and *Gpx4* ^Mac‐KO^ mice. Hematoxylin and eosin (H&E) staining revealed that adipocytes were significantly smaller in both epididymal WAT and inguinal WAT of *Gpx4* ^Mac‐KO^ mice compared to controls (Figure 4A,B). Triglyceride content was markedly reduced in both fat depots (Figure 4C,D), and cholesterol content was significantly decreased in epididymal WAT but not in inguinal WAT (Figure 4E,F).

Lipidomic analysis via LC–MS showed altered levels of several fatty acids, including myristate (14:0), palmitate (16:0), stearate (18:0), oleic acid (18:1), and linoleate (18:2), in epididymal WAT between the two groups. Notably, levels of arachidonic acid (20:4) and eicosapentaenoic acid (20:5) were significantly decreased in the epididymal WAT of *Gpx4* ^Mac‐KO^ mice compared to controls (Figure 4G). In parallel, the expression of genes related to lipid degradation—*C/EBP‐α*, *PPARγ*, *ATGL*, *Fabp4*, and *LPL*—was upregulated in epididymal WAT, while the expression of genes involved in lipid synthesis showed no significant change (Figure 4H). Similarly, *C/EBP‐α* expression was also significantly increased in inguinal WAT (Figure 4I).

Taken together, these results suggest that macrophage‐specific *Gpx4* deficiency reduces lipid accumulation and adipocyte hypertrophy, likely through enhanced lipid degradation.

### 3.5 Macrophage‐Specific *Gpx4* Deletion Limits Hepatic Steatosis in Mice

**FIGURE 5:**
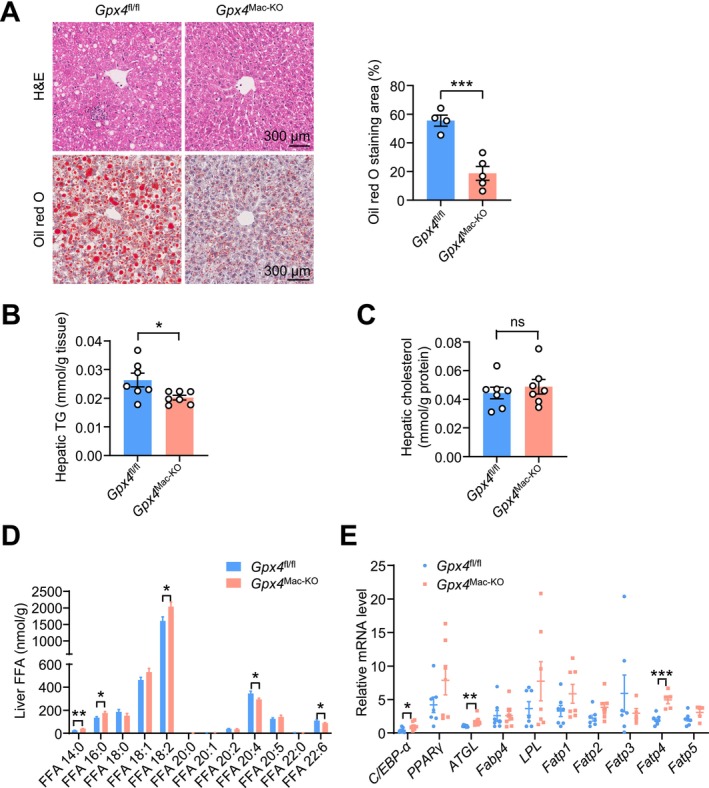
Macrophage‐specific *Gpx4* deficiency alleviates hepatic steatosis. Eight‐week‐old male *Gpx4* ^fl/fl^ and *Gpx4* ^Mac‐KO^ mice were fed on a HFD for 16 weeks. (A) H&E and Oil red O staining of livers (*n* = 4–5 per group). (B, C) Lipid content of livers (*n* = 7 per group). TG, triglycerides. (D) Levels of free fatty acids in livers collected from HFD‐fed *Gpx4* ^fl/fl^ and *Gpx4* ^Mac‐KO^ mice (*n* = 7 per group). (E) qPCR analysis of the mRNA levels of *C/EBP‐α*, *PPARγ*, *ATGL*, *Fabp4*, *LPL*, *Fatp1*, *Fatp2*, *Fatp3*, *Fatp4*, and *Fatp5* in livers (*n* = 5–7 mice per group). Data are presented as mean ± SEM. ns, not significant. *p < 0.05, **p < 0.01, ***p < 0.001 versus as indicated.

Because obesity and insulin resistance are commonly associated with hepatic steatosis, we investigated whether macrophage‐specific *Gpx4* deletion affects hepatic lipid metabolism. Hematoxylin and eosin (H&E) and Oil Red O staining revealed that HFD‐fed *Gpx4* ^fl/fl^ mice exhibited more pronounced lipid accumulation in the liver than their *Gpx4* ^Mac‐KO^ counterparts (Figure 5A). Consistently, liver triglyceride levels were significantly elevated in HFD‐fed *Gpx4* ^fl/fl^ mice compared to *Gpx4* ^Mac‐KO^ mice (Figure 5B), whereas liver cholesterol levels were comparable between the two groups (Figure 5C).

Fatty acid profiling in liver tissue revealed that levels of myristate (14:0), palmitate (16:0), and linoleate (18:2) were increased, while levels of arachidonic acid (20:4) and docosahexaenoic acid (22:6) were decreased in the livers of HFD‐fed *Gpx4* ^Mac‐KO^ mice compared with *Gpx4* ^fl/fl^ mice (Figure 5D). Correspondingly, the expression of genes involved in lipid degradation (*C/EBP‐α* and *ATGL*) and lipid transport (*Fatp4*) was significantly upregulated in the livers of *Gpx4* ^Mac‐KO^ mice (Figure 5E).

Taken together, these findings suggest that macrophage‐specific *Gpx4* deficiency alleviates HFD‐induced hepatic steatosis, potentially through enhanced fatty acid degradation and transport.

### 3.6 Ferrostatin‐1 Reverses Palmitic Acid (PA)‐induced Ferroptosis in Macrophages

Since GPX4 is a key regulator of ferroptosis, we finally investigated whether *Gpx4* deficiency‐mediated ferroptosis in macrophages is involved in the development of obesity‐associated insulin resistance by performing transcriptomic analysis on BMDMs derived from *Gpx4* ^Mac‐KO^ and *Gpx4* ^fl/fl^ mice. RNA‐sequencing (RNA‐seq) identified 637 significantly differentially expressed genes (DEGs) in *Gpx4*‐deficient BMDMs, including 360 upregulated and 277 downregulated genes (Figure S3A). Notably, approximately 80% of iron metabolism–related genes were altered in *Gpx4*‐deficient BMDMs, representing the most enriched biological process among all DEGs (Figure S3B). KEGG pathway analysis further revealed that ferroptosis‐related pathways exhibited the most substantial gene expression changes (Figure S4A).

We first verified the occurrence of ferroptosis in BMDMs from *Gpx4* ^Mac‐KO^ mice. Excessive lipid peroxidation and GSH depletion are hallmark features of ferroptosis. MDA, a major end product of lipid peroxidation, is widely used as a biochemical indicator of ferroptotic damage. Accordingly, we quantified intracellular GSH and MDA levels in BMDMs. Compared with *Gpx4* ^fl/fl^ controls, BMDMs from *Gpx4* ^Mac‐KO^ mice exhibited significantly reduced GSH levels and markedly increased MDA content (Figure S4B,C), confirming that ferroptosis occurs in BMDMs following *Gpx4* deletion.

Moreover, to directly assess macrophage ferroptosis in vivo, we performed immunofluorescence staining for F4/80 and COX2 in eWAT and iWAT. Cyclooxygenase‐2 (COX2), a well‐recognized mediator and commonly used marker of ferroptosis, was specifically evaluated within F4/80^+^ adipose tissue macrophages (ATMs). As shown in Figure S4D,E, COX2 fluorescence intensity was markedly increased in ATMs from both eWAT and iWAT of *Gpx4* ^Mac‐KO^ mice compared with *Gpx4* ^fl/fl^ controls, indicating enhanced ferroptotic activity in tissue‐resident macrophages following macrophage‐specific *Gpx4* deletion. Collectively, these findings provide direct in situ evidence for the occurrence of ferroptosis in ATMs.

**FIGURE 6:**
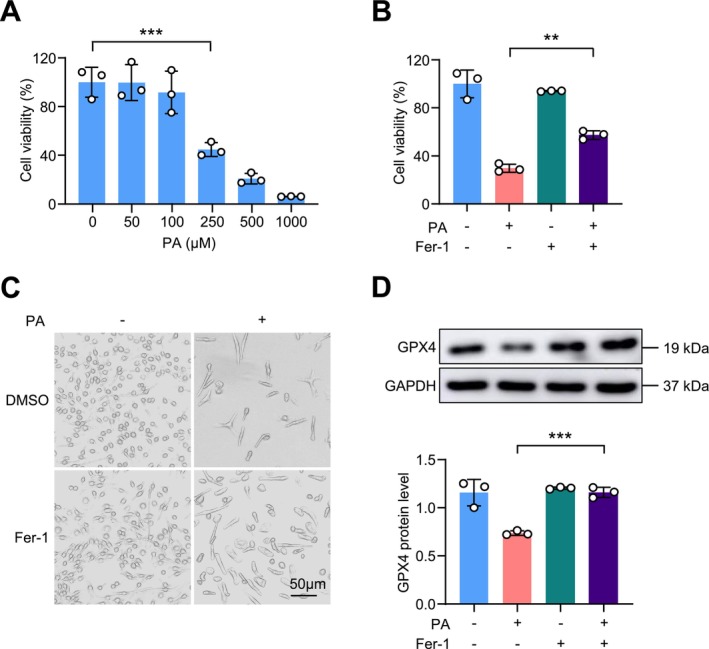
Ferroptosis inhibitor Fer‐1 can alleviate ferroptosis in BMDMs induced by palmitic acid (PA). (A) The results of the CCK8 cell viability assay after treating differentiated BMDMs isolated from C57BL/6J mice with 0, 50, 100, 250, 500, and 1000 μM of PA for 24 h (*n* = 3 mice per group). (B) Pre‐treatment of differentiated BMDMs harvested from C57BL/6J mice with Fer‐1 (6 μM) for 15 min, followed by treatment with PA (250 μM) for 24 h, and the subsequent results of the CCK8 cell viability assay (*n* = 3 mice per group). (C) A representative image of cell counts after treatment, captured under an optical microscope. (D) After subjecting BMDMs to the same treatment as depicted in B, the expression levels of GPX4 protein were detected using Western blot, and the grayscale values of the protein blots were quantified using Image J (*n* = 3 mice per group). Data are presented as mean ± SEM. **p < 0.01, ***p < 0.001 versus as indicated.

Palmitic acid (PA), the most abundant saturated long‐chain fatty acid found in the diet, is regarded as a major contributor to the development of obesity and T2D [16]. In order to mimic the obesity model in vitro, palmitic acid (PA) was used to treat macrophages. CCK8 assay showed that PA at concentrations of 250 μM and above significantly reduced BMDM viability (Figure 6A). However, treatment with Ferrostatin‐1 (Fer‐1), a potent ferroptosis inhibitor, effectively attenuated palmitic acid‐induced ferroptosis in macrophages, implying a critical role of macrophage ferroptosis in obesity‐related pathophysiology (Figure 6B,C). Furthermore, Western blot analysis showed that Fer‐1 prevented the PA‐induced reduction of GPX4 protein expression (Figure 6D). These findings suggest that PA induces ferroptosis in BMDMs and that inhibition of ferroptosis by Fer‐1 mitigates this effect, implicating macrophage ferroptosis involved in obesity.

Collectively, these findings suggest that macrophage‐specific *Gpx4* deficiency leads to widespread transcriptomic changes in ferroptosis‐ and iron metabolism–related genes in macrophages, supporting the hypothesis that ferroptosis may contribute to the mechanism by which macrophage *Gpx4* deficiency alleviates obesity and insulin resistance.

## 4 Discussion

Obesity is a global epidemic and a major contributor to the development of insulin resistance and T2D [17]. In this study, we reveal a novel role of macrophage‐specific *Gpx4* in regulating metabolic homeostasis. Mice with macrophage‐specific *Gpx4* deletion (*Gpx4* ^*Mac‐KO*^) were protected against HFD‐induced obesity and insulin resistance. These mice exhibited smaller adipocytes, reduced hepatic steatosis, and improved insulin signaling across key metabolic tissues. Biochemical assays further indicated a shift toward enhanced fatty acid catabolism and decreased systemic and tissue inflammation. Transcriptomic profiling and in vitro experiments suggested that *Gpx4* deletion‐induced ferroptosis may contribute to the improved metabolic phenotype observed in *Gpx4* ^*Mac‐KO*^ mice. Collectively, these findings demonstrate, for the first time, a link between macrophage ferroptosis and improved metabolic dysfunction, providing new insights into potential therapeutic strategies for T2D.

GPX4 is a critical antioxidant enzyme that prevents ferroptosis—a regulated form of cell death driven by iron‐dependent lipid peroxidation—by reducing lipid hydroperoxides to lipid alcohols [18]. Among all glutathione peroxidase isoforms, GPX4 is uniquely capable of suppressing ferroptosis [19]. Consistent with this, our transcriptomic analysis of bone marrow‐derived macrophages from *Gpx4* ^Mac‐KO^ mice revealed significant enrichment of ferroptosis‐associated pathways, supporting the involvement of GPX4 in regulating ferroptotic susceptibility in macrophages. While prior studies have linked *Gpx4* deletion in neurons and adipocytes to worsened metabolic outcomes [20, 21], our findings reveal that loss of GPX4 in macrophages produces the opposite effect—attenuating obesity, dyslipidemia, and insulin resistance. Moreover, *Gpx4* ^Mac‐KO^ mice exhibited lower plasma levels of LDL‐C, HDL‐C, and total cholesterol, along with improved glucose tolerance and enhanced Akt phosphorylation in the liver, adipose tissue, and skeletal muscle.

Chronic low‐grade inflammation is a hallmark of obesity and a key driver of insulin resistance and T2D [22]. Macrophages are central to this process, contributing to inflammatory cytokine production and activation of signaling pathways such as JNK/AP1 and IKK/NF‐κB [23]. The NLRP3 inflammasome further exacerbates this inflammatory milieu [24, 25]. In our study, *Gpx4* ^Mac‐KO^ mice displayed markedly reduced plasma TNF‐α and IL‐1β levels, downregulation of pro‐inflammatory genes (*Nlrp3*, *Caspase1*, and *IL‐18*), and upregulation of anti‐inflammatory markers (*Cd206*, *Fizz1*, and *Fabp4*) across key metabolic tissues. Immunohistochemistry confirmed decreased macrophage infiltration in epididymal adipose tissue. Together, these results suggest that loss of *Gpx4* in macrophages mitigates obesity‐associated inflammation, potentially through ferroptosis‐mediated depletion or functional reprogramming of inflammatory macrophages.

Our study demonstrates that macrophage ferroptosis induced by *Gpx4* deletion alleviates metabolic dysfunction and suppresses inflammation. This process is likely influenced by dynamic interactions among multiple cell types within the adipose tissue microenvironment. Emerging evidence highlights a pivotal role for Krt23^+^ fibroblasts in obesity‐associated adipose tissue inflammation. These fibroblasts secrete chemokines such as CCL2, CCL6, and CCL9, which facilitate the recruitment of pro‐inflammatory macrophages into adipose tissue [26]. Recruited M1 macrophages subsequently produce TNF‐α, which activates the NF‐κB/PHLPP1 signaling axis in glial cells, leading to glial cell apoptosis [27]. This loss of glial cell integrity compromises neurotrophic support for peripheral nerve fibers and further aggravates metabolic dysregulation. In parallel, obesity‐associated endothelial cell dysfunction—characterized by disrupted PPAR/VEGF signaling and impaired vascular barrier integrity—promotes M1 macrophage polarization and establishes a deleterious positive feedback loop between endothelial barrier disruption and macrophage‐driven inflammation. Macrophage‐derived pro‐inflammatory mediators further exacerbate endothelial dysfunction, collectively contributing to immune microenvironment imbalance within adipose tissue [28].

Obesity is also characterized by adipocyte hypertrophy and impaired lipid handling. Key enzymes responsible for triglyceride hydrolysis—adipose triglyceride lipase (*ATGL*), hormone‐sensitive lipase (*HSL*), and monoglyceride lipase (*MGL*) [29, 30, 31]—were upregulated in *Gpx4* ^Mac‐KO^ mice, alongside markers such as *C/EBP‐α*, *Pparγ*, and *Fabp4*. This was accompanied by reduced adipocyte size, lower levels of cytotoxic long‐chain saturated fatty acids, and decreased triglyceride and cholesterol content in adipose tissue, supporting the notion that *Gpx4* deletion in macrophages promotes adipose tissue lipolysis and limits lipid accumulation.

Nonalcoholic fatty liver disease (NAFLD), now redefined as metabolic dysfunction‐associated fatty liver disease (MAFLD), is tightly linked to obesity and insulin resistance [32, 33]. Since over 60% of hepatic triglycerides originate from adipose tissue lipolysis [34], the observed metabolic reprogramming in adipose tissue likely contributes to the attenuated hepatic steatosis seen in *Gpx4* ^Mac‐KO^ mice. These mice showed increased hepatic expression of lipolytic genes and decreased lipid accumulation, as evidenced by histological and Oil Red O staining. Fatty acid analysis revealed a shift toward shorter‐chain, less lipotoxic NEFAs and reduced total hepatic triglycerides, suggesting that macrophage GPX4 deletion improves hepatic lipid handling.

A notable study showed that palmitic acid (PA)‐induced cardiomyocyte death was linked to ferroptosis, and that heat shock factor 1 (HSF1) provided strong cardioprotective effects by suppressing this form of cell death [35]. We found that PA treatment decreased GPX4 levels and cell viability in vitro, which was partially rescued by the ferroptosis inhibitor ferrostatin‐1 (Fer‐1). These results indicate that PA‐induced macrophage death involves ferroptosis, thereby linking lipotoxic stress to the ferroptotic vulnerability of macrophages in obesity.

Although we provide substantial evidence supporting the protective role of macrophage‐specific *Gpx4* ablation in obesity‐induced insulin resistance and associated metabolic dysfunction, we did not determine whether inhibiting macrophage ferroptosis could reverse the beneficial effects of *Gpx4* deletion in these conditions. Further investigation is warranted.

## 5 Conclusion

Taken together, our findings establish macrophage GPX4 as a key regulator of metabolic inflammation, lipid homeostasis, and insulin sensitivity in the context of obesity. *Gpx4* deletion in macrophages protects against HFD‐induced metabolic dysfunction, potentially via ferroptosis‐associated mechanisms that promote anti‐inflammatory and lipid‐catabolic phenotypes. These results suggest that targeting macrophage ferroptosis may offer a novel therapeutic strategy for obesity‐related diseases.

## Author Contributions

S.W., X.W., H.F., S.H., and Q.W. performed the experiments, collected, and analyzed data. S.W., X.W., J.P., H.G., and Y.D. contributed to manuscript writing. H.T. edited the manuscript. M.Z., J.P., Y.L., and G.Z. contributed to the scientific discussion. X.D. supervised the study reviewed and edited the manuscript, and acquired funding. All authors contributed to the article and approved the submitted version.

## Funding

This work was supported by MOST | National Natural Science Foundation of China (NSFC), 82570537 and 82370462; Key Project of the Hunan Province Natural Science Foundation University Joint Fund, 2025JJ90126; Beijing Natural Science Foundation‐Changping Innovation Joint Fund, L254078; Foundation of Hunan Provincial Key Laboratory, 2023TP1014; Hunan Provincial Clinical Medical Research Center for Obesity‐Related Diseases, 2023SK4052; The Joint Medical and Health Sector Program of the Hunan Provincial Natural Science Foundation, 2025JJ81004.

## Ethics Statement

All animal experiments were approved by the Ethics Committee of Guangzhou Medical University (GY2019‐016) and conducted in accordance with relevant international, national, and institutional guidelines for animal care and use.

## Conflicts of Interest

The authors declare no conflicts of interest.

## Supporting information


**Table S1:** fsb271427‐sup‐0001‐TableS1.docx.

**Figures S1–S4:** fsb271427‐sup‐0002‐FiguresS1‐S4.docx.

## Data Availability

The data that support the findings of this study are available in the original Western blots and figures.
