# Supplementary material for: Gpx4 Deletion‐Mediated Macrophage Ferroptosis Alleviates Obesity‐Associated Insulin Resistance
Source: FASEB J. 2026 Jan 12;40(2):e71427. doi: 10.1096/fj.202503596R (PMC12794132; doi:10.1096/fj.202503596R)
Supplement: Supplementary file 1 — Table S1: fsb271427‐sup‐0001‐TableS1.docx. [file FSB2-40-e71427-s002.docx]

**Supplementary Materials**

***Gpx4* deletion-mediated macrophage ferroptosis alleviates obesity-associated insulin resistance**

**Table S1.**

**Table S1.** List of primers used for quantitative real-time PCR (qPCR).

| Gene  (Mouse) | Forward primer sequence | Reverse primer sequence |
| --- | --- | --- |
| *Nlrp3* | ATTACCCGCCCGAGAAAGG | TCGCAGCAAAGATCCACACAG |
| *ASC* | CTTGTCAGGGGATGAACTCAAAA | GCCATACGACTCCAGATAGTAGC |
| *Caspase1* | ACAAGGCACGGGACCTATG | TCCCAGTCAGTCCTGGAAATG |
| *IL-18* | TCAAAGTGCCAGTCACCCC | GGTCACGCCAGTCCTCTTAC |
| *IL-1β* | CTCGTGCTGTCGGACCCAT | CAGGCTTGTGCTCTGCTTGTGA |
| *TNF-α* | GTTCTATGGCCCAGACCCTCACA | TACCAGGGTTTGAGCTCAGC |
| *Emr1* | CCTGGACGAATCCTGTGAAG | GGTGGGACCACAGAGAGTTG |
| *Ccl2* | CATCCACGTGTTGGCTCA | GATCATCTTGCTGGTGAATGAGT |
| *Arg2* | TGATTGGCAAAAGGCAGAGG | CTAGGAGTAGGAAGGTGGTC |
| *CD206* | CAAGTGATTTGGAGGCT | TAGGAAACGGGAGAACC |
| *Fabp4* | TGATGCCTTTGTGGGAACCT | TTGTGGTCGACTTTCCATCCC |
| *Mgl2* | AGGCACCCTAAGAGCCATTT | CCCTCTTCTCCAGTGTGCTC |
| *Fizz1* | TCGTGGAGAATAAGGTCAAGG | AGGAGGCCCATCTGTTCATA |
| *Ym1* | ACCAGGAAAGTACACAGATGA | CACGGCACCTCCTAAATTGT |
| *iNOS* | GAGCCAGTTGTGCATTGTC | CCAGGAAGTAGGTGAGGG |
| *β-actin* | ATCTGGCACCACCTTC | AGCCAGGTCCAGACGCA |
| *C/EBP-α* | CTAGGAGATTCCGGTGTGGC | CCCGAGAGGAAGCAGGAATC |
| *PPARγ* | GTGAGACCAACAGCCTGACG | CTTCCATCACGGAGAGGTCC |
| *ATGL* | GACAGCTCCACCAACATCCA | GAGGCGGTAGAGATTGCGAA |
| *LPL* | GCCTTTCTCCTGATGACGCT | AACTCAGGCAGAGCCCTTTC |
| *FAS* | GAGGACACTCAAGTGGCTGA | GTGAGGTTGCTGTCGTCTGT |
| *Srebp-1c* | GGAGCCATGGATTGCACATT | GGCCCGGGAAGTCACTGT |
| *ACC* | TGTACAAGCAGTGTGGGCTGGCT | CCACATGGCCTGGCTTGGAGGG |
| *PLIN* | CCCGGCTCTTCAATACCCTC | TGGTGGCAGGAGGAACTCTA |
| *Fatp1* | CGCTTTCTGCGTATCGTCTGCAAG | AAGATGCACGGGATCGTGTCT |
| *Fatp2* | CTGATGATCGACCGTGAGAA | TACCAGTCCCACGATGTCAG |
| *Fatp3* | AGGCTGCTCGAATCAGTCAT | AACTTGGGTTTCAGCACCAC |
| *Fatp4* | CAGCAACTGTGACCTGGAGA | CCTTCCGCAACTCTGTCTTC |
| *Fatp5* | GGTTTTTGCATTCCTGTGGA | GAAGGGTTGGTTCTTTCGAA |
| *Gpx4* | CCTCTGCTGCAAGAGCCTCCC | CTTATCCAGGCAGACCATGTGC |
| *GAPDH* | ATTGTCAGCAATGCATCCTG | ATGGACTGTGGTCATGAGCC |
