# Supplementary material for: Gpx4 Deletion‐Mediated Macrophage Ferroptosis Alleviates Obesity‐Associated Insulin Resistance
Source: FASEB J. 2026 Jan 12;40(2):e71427. doi: 10.1096/fj.202503596R (PMC12794132; doi:10.1096/fj.202503596R)
Supplement: Supplementary file 2 — Figures S1–S4: fsb271427‐sup‐0002‐FiguresS1‐S4.docx. [file FSB2-40-e71427-s001.docx]

**Supplementary Materials**

***Gpx4* deletion-mediated macrophage ferroptosis alleviates obesity-associated insulin resistance**

**Figure S1-S4**

**Figure S1**





**Figure S1. Generation and validation of** **macrophage-specific *Gpx4* knockout (*Gpx4*^Mac-KO^) mice.**

(A) Schematic diagram of the breeding strategy for generating *Gpx4*^Mac-KO^ mice. (B) Representative PCR-based genotyping. *Gpx4*^fl/fl^/*LysM*-Cre*^−^* and *Gpx4*^fl/fl^/*LysM*-Cre*^+^* mice are designated as *Gpx4*^fl/fl^ (control) and *Gpx4*^Mac-KO^, respectively. (C, D) qPCR analysis of *Gpx4* mRNA expression in peritoneal macrophages (PMs, C) and bone marrow-derived macrophages (BMDMs, D) from *Gpx4*^fl/fl^ and *Gpx4*^Mac-KO^ mice (n = 3 mice per group). (E and F) Immunoblot analysis of GPX4 protein levels in PMs (E) and BMDMs (F) from *Gpx4*^fl/fl^ and *Gpx4*^Mac-KO^ mice (n = 3–4 mice per group). Data are presented as mean ± SEM. **P* < 0.05, ***P* < 0.01, ****P* < 0.001, *****P* < 0.0001 versus indicated groups.

Figure S2





Figure S2. **Macrophage-specific *Gpx4* deficiency does not affect body weight or insulin sensitivity under a chow diet. Eight-week-old male *Gpx4*^fl/fl^ (n = 7) and *Gpx4*^Mac-KO^ (n = 8) mice were fed a chow diet for 16 weeks.** (A) Body weight was measured biweekly from 8 to 24 weeks of age. (B) Representative images of *Gpx4*^fl/fl^ and *Gpx4*^Mac-KO^ mice. (C) Representative images (top) and quantification (bottom) of fat pads, including epididymal white adipose tissue (eWAT), perirenal WAT, inguinal WAT, and brown adipose tissue (BAT). (D and E) Glucose tolerance test (GTT) and insulin tolerance test (ITT). Data are presented as mean ± SEM. ns, not significant.

**Figure S3**





**Figure S3. Transcriptomic profiling of BMDMs from *Gpx4*^fl/fl^ and *Gpx4*^Mac-KO^ mice. BMDMs were isolated and differentiated using M-CSF.** (A) Volcano plot showing significantly differentially expressed genes in BMDMs (n = 3). (B) Gene Ontology (GO) enrichment analysis of altered genes, highlighting changes in iron metabolism–related pathways.

**Figure S4**





**Figure S4. Ferroptosis in BMDMs and white adipose tissue macrophages upon macrophage-specific *Gpx4* deletion.** (A) BMDMs from *Gpx4*^fl/fl^ and *Gpx4*^Mac-KO^ mice were extracted and differentiated using M-CSF, followed by RNA-seq analysis. KEGG pathway enrichment analysis of differentially expressed genes in *Gpx4*-deficient BMDMs. (B) GSH level in BMDMs of *Gpx4*^fl/fl^ and *Gpx4*^Mac-KO^ mice (n = 3 mice per group). (C) MDA level in BMDMs of *Gpx4*^fl/fl^ and *Gpx4*^Mac-KO^ mice (n = 3 mice per group). (D, E) Immunofluorescence staining of F4/80 and COX2 in eWAT and iWAT of HFD-fed *Gpx4*^fl/fl^ and *Gpx4*^Mac-KO^ mice. Data are presented as mean ± SEM. ^*^*P* < 0.05, ^**^*P* < 0.01, ^***^*P* < 0.001 versus as indicated.
